# The Role of Prostate Apoptosis Response-4 (Par-4) in *Mycobacterium tuberculosis* Infected Macrophages

**DOI:** 10.1038/srep32079

**Published:** 2016-08-24

**Authors:** Ji-Ye Han, Yun-Ji Lim, Ji-Ae Choi, Jung-hwan Lee, Sung-Hee Jo, Sung-Man Oh, Chang-Hwa Song

**Affiliations:** 1 Department of Medical Science, 266 Munhwa-ro, Jung-gu, Daejeon, 35015, Republic of Korea; 2 Department of Microbiology, 266 Munhwa-ro, Jung-gu, Daejeon 35015, Republic of Korea; 3 Research Institute for Medical Sciences, College of Medicine, Chungnam National University, 266 Munhwa-ro, Jung-gu, Daejeon 35015, Republic of Korea

## Abstract

Prostate apoptosis response-4 (Par-4) is a tumor suppressor protein that forms a complex with glucose-regulated protein 78 (GRP78) to induce apoptosis. Previously, we reported that ER stress-induced apoptosis is a critical host defense mechanism against *Mycobacterium tuberculosis (Mtb*). We sought to understand the role of Par-4 during ER stress-induced apoptosis in response to mycobacterial infection. Par-4 and GRP78 protein levels increased in response *Mtb* (strain: H37Ra) infection. Furthermore, Par-4 and GRP78 translocate to the surface of *Mtb* H37Ra-infected macrophages and induce apoptosis via caspase activation. NF-κB activation*, Mtb*-mediated ER stress, and Par-4 production were significantly diminished in macrophages with inhibited ROS production. To test Par-4 function during mycobacterial infection, we analyzed intracellular survival of *Mtb* H37Ra in macrophages with Par-4 overexpression or knockdown. *Mtb* H37Ra growth was significantly reduced in Par-4 overexpressing macrophages and increased in knockdown macrophages. We also observed increased Par-4, GRP78, and caspases activation in Bacillus Calmette-Guérin (BCG)-infected prostate cancer cells. Our data demonstrate that Par-4 is associated with ER stress-induced apoptosis resulting in reduced intracellular survival of mycobacteria. BCG treatment increases Par-4-dependent caspase activation in prostate cancer cells. These results suggest ER stress-induced Par-4 acts as an important defense mechanism against mycobacterial infection and regulates cancer.

Immunotherapy with mycobacteria has been used successfully as a treatment for bladder and prostate cancers^1^,^2^. Bacillus Calmette-Guérin (BCG) is a strain of *Mycobacteria bovis* that effectively induces an immune response against tumor cells, leading to a reduction of risk factors associated with tumor recurrence and progression^1^,^3^. However, the exact mechanisms of mycobacterial immunotherapy are not fully understood.

Prostate apoptosis response-4 (Par-4) is a protein that was first identified in prostate cancer cells undergoing apoptosis^4^,^5^. Although Par-4 is incapable of directly inducing apoptosis in normal cells, Par-4 overexpression is sufficient to induce apoptosis in most cancer cells^6^. Under normal conditions, Par-4 resides predominantly in the cytosol, but is secreted out of the cell through the plasma membrane in response to apoptotic stress^5^. Secretion of Par-4 follows the conventional ER-Golgi secretory pathway, and is associated with ER stress^5^. Fascinatingly, secreted Par-4 can induce cancer cell-specific apoptosis^5^,^6^.

Macrophages are the first line of innate immune defense against mycobacteria. Induction of apoptosis in infected macrophages is considered a host-protective response that decreases bacterial load and intracellular bacterial survival^7^. Many studies suggest that regulation of apoptosis in mycobacterial-infected cells may be an important mechanism for the removal of intracellular mycobacteria^8^,^9^,^10^. Our recent findings demonstrated that ER stress-mediated apoptosis in macrophages is important for reducing intracellular survival of mycobacteria^11^. Par-4 secretion is mediated by GRP78 (glucose-regulated protein 78)^12^. GRP78 is a major ER chaperone protein involved in many cellular processes that contribute to normal function of the ER^12^. A recent study suggests that GRP78 restores cells from stress conditions following *Chlamydia pneumonia* infection. However, there is little known about the role of GRP78 during mycobacterial infection^13^.

We hypothesize that *Mtb*-induced GRP78 production coordinates with Par-4 to induce apoptosis of *Mtb*-infected macrophages. In this study, we demonstrated that *Mtb*-induced ER stress increased the production of Par-4 in macrophages, resulting in suppression of intracellular mycobacterial survival through the induction of apoptosis.

## Results

### *Mtb* H37Ra-induced Par-4 production is associated with ER stress in macrophages

**Figure 1:**
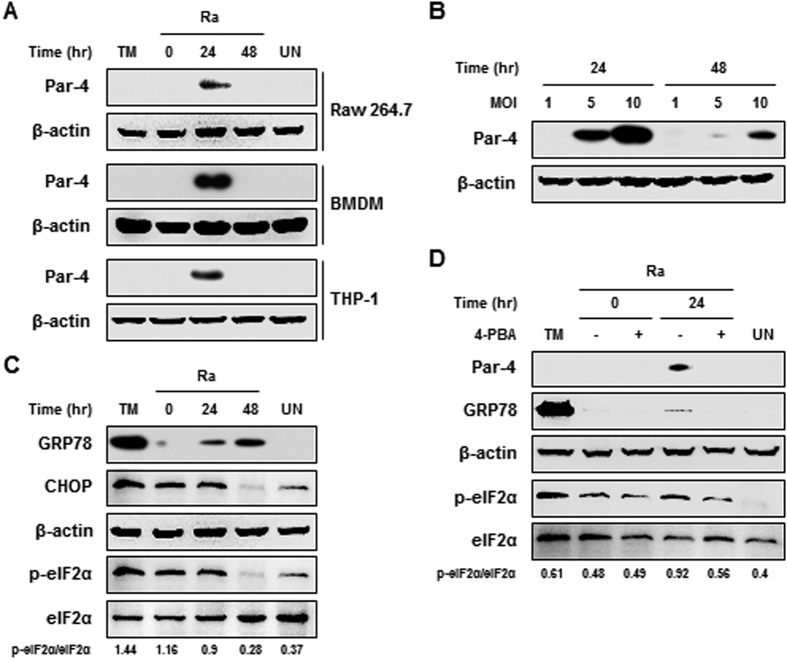
*Mtb* H37Ra-induced Par-4 production is associated with ER stress in macrophages. (**A**) RAW 264.7 cells, BMDM, and THP-1 cells were infected with *Mtb* H37Ra at an MOI of 5, and incubated for 0–48 h. Par-4 expression was analyzed by Western blot using a Par-4 specific antibody. (**B**) RAW 264.7 cells were infected with at MOI of 1, 5 and 10, and incubated for indicated times. (**C**) ER stress sensor molecules in H37Ra (MOI = 5)-infected macrophages were detected by Western blot analysis. (**D**) RAW 264.7 cells were pretreated with 4-PBA (5 μM) for 1 h and then infected with H37Ra for 24 h. Tunicamycin (TM; 2 μg/mL) was used as a positive control for ER stress. Data are presented as the mean ± SD of three independent experiments.

To determine whether *Mtb* induces Par-4 production in macrophages, we measured Par-4 levels by Western blot analysis of RAW 264.7 macrophages infected with the H37Ra strain of *Mtb*. Par-4 was increased 24 h after mycobacterial infection in RAW 264.7 cells. Similar results were obtained using bone marrow-derived macrophages (BMDM) and THP-1 monocytes infected with *Mtb* H37Ra (Fig. 1A). Par-4 production increased with a greater (multiplicity of infection) MOI (Fig. 1B). These data suggest that *Mtb* H37Ra infection induces production of Par-4 in macrophages.

Intracellular Par-4 binds to the ER stress monitor, GRP78, and is translocated to the cell surface^6^. We found that ER stress sensor molecules, including phospho-eIF2α, GRP78, and CHOP were induced by *Mtb* H37Ra infection (Fig. 1C). The production of phospho-eIF2α increased immediately in RAW 264.7 macrophages after H37Ra infection. Additionally, the production of GRP78 and CHOP increased gradually for 48 h after H37Ra infection (Fig. 1C).

**Figure 2:**
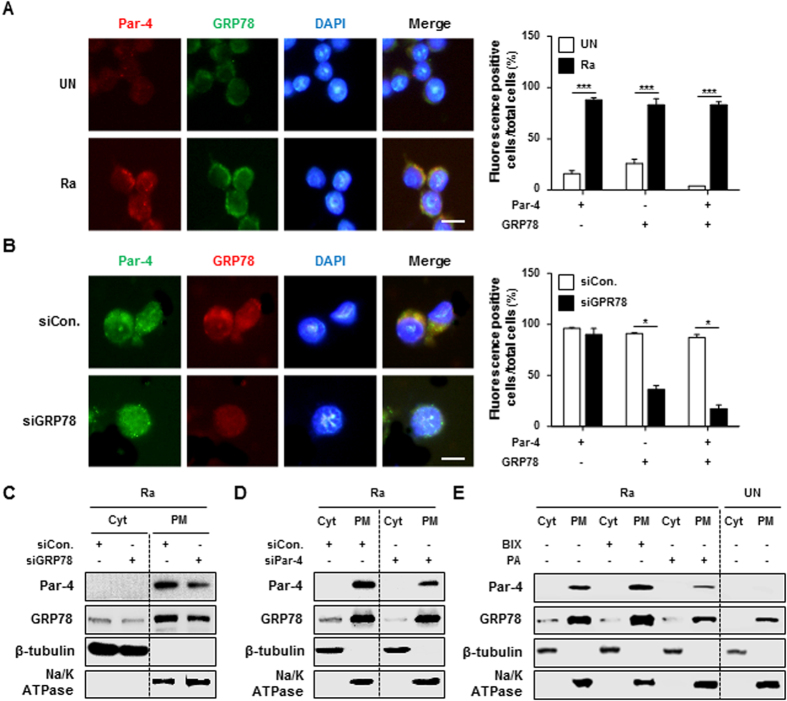
GRP78 plays an essential role in Par-4 translocation to the plasma membrane of macrophages during *Mtb* H37Ra infection. (**A**) RAW 264.7 cells were infected with *Mtb* H37Ra at an MOI of 10 for 24 h. Par-4 and GRP78 localization to plasma membrane were confirmed by immunofluorescence staining. (**B**) RAW 264.7 cells were transfected with control siRNA (200 nM) or GRP78 siRNA (200 nM) and incubated overnight. Transfected macrophages were infected with H37Ra (MOI = 10) for 24 h. Immunofluorescence staining was performed using specific antibodies against Par-4 (green), GRP78 (red), and DAPI (blue). Colocalization of Par-4 and GRP78 was shown in merged images (yellow). Number of Par-4^+^ cells, GRP78^+^ cells, and Par-4^+^GRP78^+^ cells were counted manually. The stained cells were identified by fluorescence microscopy (400×). Statistically significant differences are indicated by *p < 0.05, **p < 0.01, ***p < 0.001. RAW 264.7 cells were transfected overnight with (**C**) GRP78 siRNA (200 nM) or (**D**) Par-4 siRNA (200 nM). Then, cells were infected with H37Ra (MOI = 5) for 24 h. Expression of Par-4 and GRP78 were detected by Western blot. (E) RAW 264.7 cells were pretreated with BiP inducer X (BIX; 5 μM) and Piericidin A (PA; 500 nM) for 1 h, after infected with H37Ra (MOI = 5) for 24 h. Whole cells were fractionated into cytoplasm fractions and plasma membrane fractions, and detected expression of Par-4 and GRP78 by Western blot analysis. The β-tubulin (cytoplasm) and Na^+^/K^+^ ATPase (plasma membrane) were used as loading controls for cell lysates. Cyt: cytoplasm, PM: plasma membrane. Data are presented as the mean ± SD of three independent experiments.

To determine whether ER stress is associated with production of Par-4 (see Supplementary Figure 1 online), we examined Par-4 production in *Mtb* H37Ra-infected macrophages treated with the chemical chaperone and inhibitor of ER stress, 4-phenylbutyrate (4-PBA). Our Western blot analysis demonstrated that *Mtb* induced Par-4 production was significantly reduced by 4-PBA (Fig. 1D). Since Par-4 localization is associated with the regulation of cell death, we used immunofluorescence to determine whether Par-4 translocates to the cell surface during *Mtb* infection. Translocation of Par-4 and GRP78 from the cytoplasm to the plasma membrane was increased in macrophages 24 h post mycobacterial infection (Fig. 2A). To investigate the role of GRP78 in Par-4 translocation, we visualized Par-4 localization in *Mtb*-infected macrophages after knockdown of GRP78 by a specific siRNA. Translocation of Par-4 to the plasma membrane was significantly decreased in the GRP78 siRNA knockdown compared with control siRNA treated cells (Fig. 2B). The redistribution of Par-4 to the plasma membrane was reduced in GRP78 siRNA-transfected macrophages and Par-4 siRNA-transfected macrophages, as confirmed by Western blot analysis (Fig. 2C,D). Localization of Par-4 to the plasma membrane was increased by the GRP78 inducer, BiP protein inducer X (BIX) (Fig. 2E). In contrast, treatment with Piericidin A, a GRP78 inhibitor, reduced localization of Par-4 to the plasma membrane (Fig. 2E). Together, these data indicate that GRP78 plays a key role in the translocation of Par-4 from the cytoplasm to the plasma membrane during *Mtb* infection.

### Up-regulated Par-4 leads to apoptosis in *Mtb*-infected macrophages

**Figure 3:**
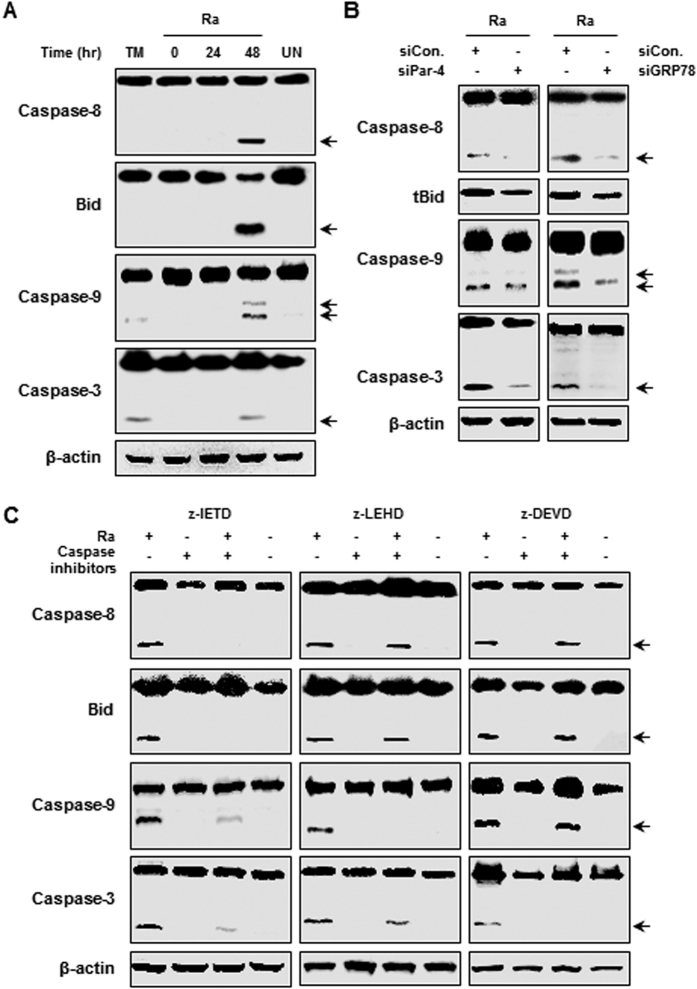
*Mtb* H37Ra-mediated Par-4 production induces caspase-dependent apoptosis. (**A**) RAW 264.7 cells infected with H37Ra (MOI = 5) were confirmed activation of caspase-8, BID, caspase-9 and caspase-3 by Western blot analysis. (**B**) RAW 264.7 cells were transfected with control siRNA (200 nM), Par-4 siRNA (200 nM) and GRP78 siRNA (200 nM) for overnight, infected *Mtb* H37Ra (MOI = 5) for 24 h and then analyzed activation of caspase-8, tBID, caspase-9 and caspase-3 by Western blot. (**C**) RAW 264.7 cells were transfected with control siRNA (200 nM) and Par-4 siRNA (200 nM) prior to H37Ra infection for 24 h. These cells were fractionated into cytoplasm and mitochondria, and cytochrome C release was analyzed by Western blot. For cytosolic and mitochondrial loading control, β-tubulin and COX IV were used, respectively. Data are presented as mean ± SD of three independent experiments.

Binding of Par-4 with GRP78 on the cell surface of macrophages induces apoptosis through caspase activation^5^. In order to determine the functional relationship between Par-4 translocation and apoptosis, we examined the relationship between Par-4 production and activation of caspases during *Mtb* H37Ra infection. We found that caspase-8 activation by *Mtb* infection initiated cleavage of BID into tBID, which prompted cleavage and activation of caspase-9 and -3, leading to apoptosis (Fig. 3A). Levels of cleaved caspases, including caspase-8, -9, and -3, were remarkably decreased in Par-4 or GRP78 siRNA-transfected macrophages compared with control siRNA-transfected macrophages (Fig. 3B). These data suggest that Par-4-mediated apoptosis in macrophages is induced by activation of the caspases during *Mtb* H37Ra infection and is mediated by GRP78.

To define crosstalk between caspase-8 and -9 activation, we analyzed the activation of caspases using specific inhibitors of caspase-8, -9, and -3. The cleavage of BID and activation of caspase-9 and -3 were reduced in macrophages pretreated with the caspase-8 specific inhibitor (Z-IETD-FMK) prior to *Mtb*-infection compared with untreated cells (Fig. 3C). Interestingly, caspase-8 activation and tBID induction by *Mtb* infection was not significantly different in macrophages treated with the caspase-9 specific inhibitor (Z-LEHD-FMK) or caspase-3 specific inhibitor (Z-DEVD-FMK). These data show that caspase-8 activation during *Mtb* infection controls downstream tBID expression following activation of caspase-9 and -3. Thus, *Mtb*-induced Par-4 production plays a key role in activating the caspase signaling cascade.

### *Mtb* infection activates NF-κB and promotes Par-4 production

**Figure 4:**
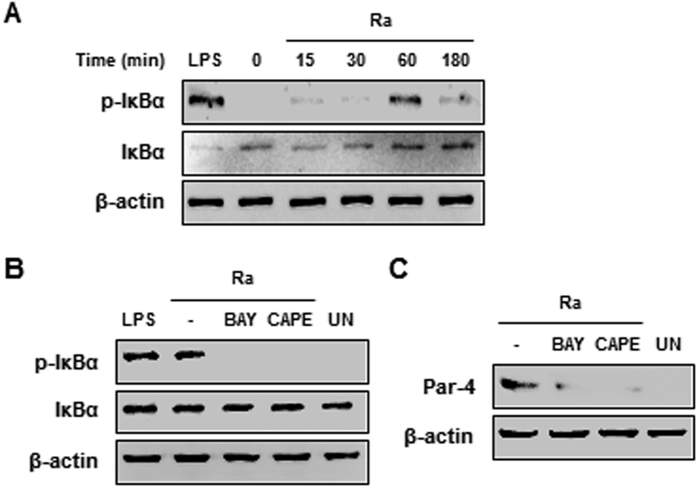
NF-κB activation is essential for *Mtb* H37Ra-induced Par-4 expression. (**A**) RAW 264.7 cells were infected with *Mtb* H37Ra (MOI = 5), and incubated for 0–180 min. Total IκBα and p-IκBα were detected by Western blot using specific antibodies. (**B**) RAW 264.7 cells were pretreated with BAY 11-7085 (BAY; 1 μM) or caffeic acid phenethyl ester (CAPE; 1 μM) for 1 h and then infected with *Mtb* H37Ra (MOI = 5). Expression of IκBα and p-IκBα was confirmed by Western blot analysis. (**C**) RAW264.7 cells pretreated BAY or CAPE were infected with *Mtb* H37Ra for 24 h, and analyzed Par-4 expression. Data are presented as mean ± SD of three independent experiments.

Regulation of the NF-κB signaling pathway is important for the control of *Mtb* infection^14^. It has been reported that NF-κB has pro- and anti-apoptotic effects depending on the stimulus^15^. In order to investigate the role of NF-κB activation in Par-4-induced apoptosis, we examined the phosphorylation and degradation of IκBα, which sequesters inactive NF-kB dimers in the cytoplasm, in *Mtb* H37Ra-infected macrophages by immunoblot analysis. Phosphorylated-IκBα was increased 60 min after *Mtb* infection in RAW 264.7 cells (Fig. 4A). NF-κB inhibitors, BAY 11-7085 (BAY) and caffeic acid phenethyl ester (CAPE), caused a significant reduction to Par-4 production and IκBα phosphorylation in *Mtb*-infected macrophages (Fig. 4B,C). These data suggest that NF-κB activation promotes Par-4 expression in *Mtb*-infected macrophages, leading to apoptosis.

### Intracellular ROS generation is important to Par-4-mediated apoptosis during *Mtb* H37Ra infection

**Figure 5:**
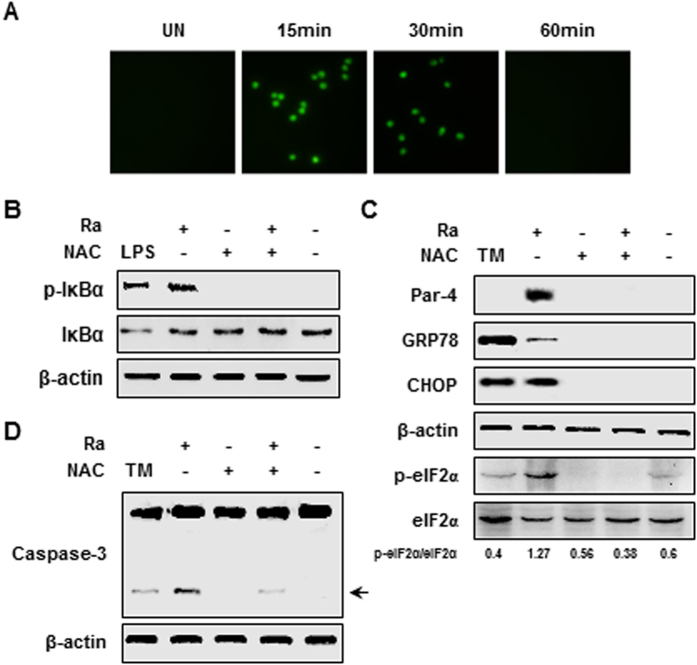
NF-κB-induced Par-4 activation is required intracellular ROS generation by *Mtb* H37Ra infection. (**A**) RAW 264.7 cells were infected with *Mtb* H37Ra (MOI = 5) for 0–60 min and intracellular ROS production was evaluated by DCFDA (5 μM) staining. RAW 264.7 cells were pretreated with ROS scavenger (NAC; 20 mM) for 1 h and then infected with H37Ra (MOI = 5). (**B**) NF-κB activation at 1 h and (**C**) expression of Par-4 and ER stress sensor molecules were measured 24 h after H37Ra infection. (**D**) After 48 h of infection, apoptotic cell death was indicated by the presence of cleaved form of caspase-3 by Western blot. Lipopolysaccharide (LPS; 500 ng/mL) was used for positive control for NF-κB activation. Data are presented as mean ± SD of three independent experiments.

Recent studies have shown that *Mtb* infection increases ROS generation in macrophages^11^,^16^. Moreover, Par-4 production is dependent on ROS generation^17^. To investigate the effects of *Mtb*-induced ROS generation on Par-4 production, we measured intracellular ROS production in *Mtb*-infected macrophages using dichloro-dihydro-fluorescein diacetate (DCFH-DA) staining. Intracellular ROS levels were elevated within 30 min of *Mtb* infection (Fig. 5A). Pretreatment with the ROS inhibitor, N-acetyl-L-cysteine (NAC), following *Mtb* infection significantly decreased NF-κB activation in macrophages (Fig. 5B). Since ROS production is closely associated with ER stress-induced apoptosis in *Mtb* infected macrophages, we assessed production of ER stress sensor molecules (GRP78, eIF2α, and CHOP) and caspase-3 activation in NAC-pretreated macrophages. *Mtb*-mediated ER stress and Par-4 production were significantly diminished in macrophages with inhibited ROS production (Fig. 5C,D) indicating that *Mtb*-mediated ER stress is important to induce ROS generation, leading to Par-4-mediated apoptosis.

### Effects of Par-4 expression on intracellular survival of *Mtb* H37Ra

**Figure 6:**
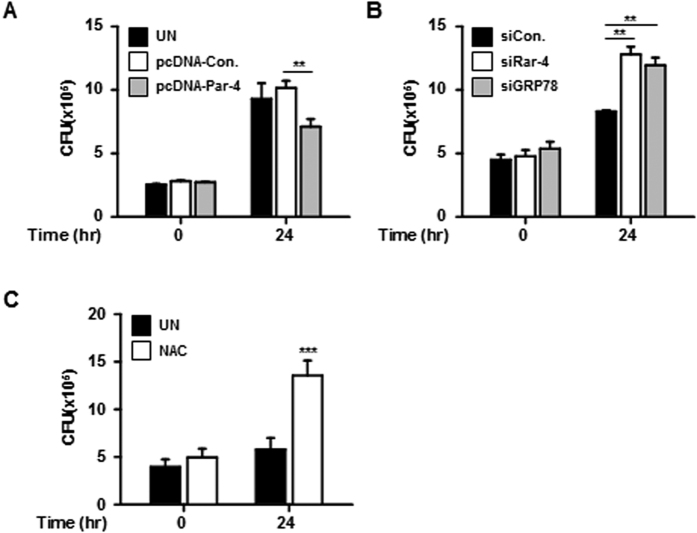
Par-4 regulates intracellular survival of *Mtb* H37Ra in macrophages. The intracellular survival of *Mtb* H37Ra (MOI = 5) in RAW 264.7 cells was quantified by CFU analysis. (**A**) RAW 264.7 cells overexpressed pcDNA3.1 (1 μg/mL) and pcDNA3.1-Par-4 (1 μg/mL) and then were infected with *Mtb* H37Ra for 24 h. (**B**) RAW 264.7 cells were transfected with control siRNA (200 nM) and Par-4 siRNA (200 nM) prior to H37Ra infection for 24 h. (**C**) RAW 264.7 cells pretreated with NAC (20 nM) were infected with H37Ra for 24 h. Statistically significant differences are indicated by *p < 0.05, **p < 0.01, ***p < 0.001. Data are presented as mean ± SD of three independent experiments.

To investigate whether Par-4 production affects the intracellular survival of *Mtb* in macrophages, we evaluated the intracellular growth of *Mtb* H37Ra in Par-4 overexpressing or knockdown macrophages. Intracellular survival of *Mtb* was significantly reduced in Par-4 overexpressing macrophages compared with the control (Fig. 6A). In contrast, intracellular survival of *Mtb* was increased in Par-4 or GRP78 knockdown macrophages (Fig. 6B). Interestingly, survival of *Mtb* was significantly increased in NAC-pretreated macrophages compared with control cells (Fig. 6C). Taken together, these data suggest that ROS-dependent production of Par-4 regulates intracellular survival of *Mtb*.

### Par-4-dependent apoptosis is a novel mechanism in the control of tumor cell viability

**Figure 7:**
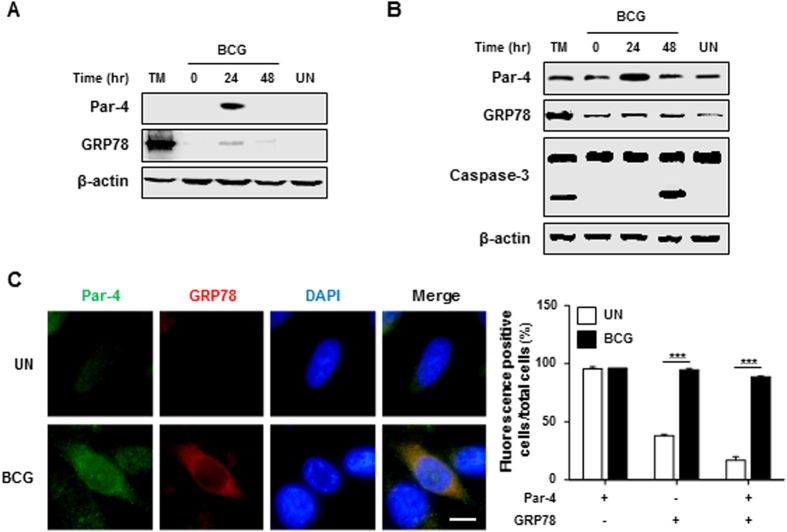
BCG treatment induces Par-4-dependent apoptosis in PC-3 cells. (**A**) RAW 264.7 cells or (**B**) PC-3 cells were infected with BCG (MOI = 5) for 3 h and then incubated for 0–48 h. Whole cell lysates were prepared and activation of Par-4, GRP78 and caspase-3 were detected using specific antibodies in Western blot analysis. (**C**) PC-3 cells were infected with BCG (MOI = 10) for 24 h, and localization of Par-4 and GRP78 were confirmed by immunofluorescence staining using specific antibodies against Par-4 (green), GRP78 (red) and DAPI (blue). Statistically significant differences are indicated by *p < 0.05, **p < 0.01, ***p < 0.001. Data are presented as mean ± SD of three independent experiments.

Since Par-4 is remarkably decreased in cancer tissues^4^ and BCG immunotherapy has been used successfully as a treatment modality for prostate and bladder cancer^18^, we hypothesized that Par-4 induction by mycobacterial infection may contribute to the inhibition of bladder cancer cell growth. To confirm this hypothesis, we evaluated Par-4 and GRP78 protein levels in *M. bovis* BCG-infected macrophages. Production of Par-4 and GRP78 was increased in *Mtb*-infected RAW 264.7 cells 24 h post-infection (Fig. 7A). Interestingly, production of Par-4 and GRP78, as well as caspase-3 activation was increased in human prostate cancer cell lines (PC-3) after *M. bovis* BCG infection (Fig. 7B). Additionally, we found that enhanced level of Par-4 was co-localized with GRP78 at plasma membrane in PC-3 prostate cancer cells treated with *M. bovis* BCG (Fig. 7C). These data suggest that BCG-treated PC-3 cells induce Par-4 production, which promotes apoptotic cell death of PC-3 prostate cancer cells.

## Discussion

During *Mtb* infection, apoptosis of macrophage is an important host defense mechanism to eliminate intracellular mycobacteria^19^,^20^. In the present study, we show that Par-4 translocation to the plasma membrane is dependent on ER stress-mediated apoptosis in macrophages during *Mtb* infection. Par-4 has been shown to be a regulator of apoptosis in prostate cancer cells^4^,^5^ by inducing apoptosis selectively in tumor cells, but not in normal cells^4^,^5^. Our results show that Par-4 expression is closely associated with ER stress-mediated apoptosis during *Mtb* infection. Similarly, a previous report showed that Par-4 mediated neuronal apoptosis in HIV encephalitis^21^. Under ER stress conditions, Par-4 binds to endogenous GRP78 through its SAC (selective for apoptosis in cancer cells) domain. Then, Par-4/GRP78 complex is translocated from the cytoplasm to cell surface and Par-4 is spontaneously secreted into the serum^22^. We demonstrated that Par-4 translocation was affected by mycobacteria-mediated GRP78 production in macrophages. *Mtb*-induced GRP78 raised the possibility that it binds to Par-4, and GRP78/Par-4 complex is localized at plasma membrane of macrophage. Previously, we suggested that ER stress induced by infection or antigen stimulation of mycobacteria is important to regulate apoptotic cell death^11^,^23^,^24^. GRP78 localized to the plasma membrane acts as a receptor for extracellular Par-4 leading to activation of apoptosis via mitochondrial release of cytochrome C and caspase activation^25^.

We demonstrated that Par-4-mediated apoptosis increased during *Mtb* infection. Additionally, *Mtb*-induced caspase-8 activation regulates BID cleavage, triggering activation of caspase-9 and -3. It was reported that extracellular Par-4 activates caspase-8 through recruitment of FAS-associated death domain protein (FADD)^25^, while caspase-8 cleaves intracellular Par-4. Then, only the cleaved C-terminus of Par-4 enters the nucleus^26^. When expression of Par-4 or GRP78 was knocked down with specific siRNA, caspase activation was significantly decreased (Fig. 3B). Activated caspase-8 leads to mitochondrial damage through cleavage of BID to tBID and then caspase-9 activation^27^,^28^. These data indicate that the interaction between Par-4 and GRP78 is important for inducing apoptosis by caspase-8 activation during mycobacterial infection.

NF-κB binds to the Par-4 promoter region and enhances its transcriptional activation^15^. We have shown that ROS generation is important for inducing Par-4 production via the activation of NF-κB and ER stress during *Mtb* infection. NF-κB activation is not only associated with Par-4 production, but also with infection control^14^,^15^. Importantly, ROS induce ER stress and lead to NF-κB activation during mycobacterial infection^23^,^24^. These data suggest that mycobacteria-mediated ROS generation is important for inducing Par-4 mediated apoptosis through ER stress activation.

These findings are important because we demonstrate that ER stress acts as a host defense mechanism by inducing Par-4 production to reduce intracellular survival of mycobacteria. Moreover, our results provide insight on how BCG immunotherapy is effective against prostate cancer cells. Par-4 has been established as a potential target for cancer cell-selective pro-apoptotic protein^5^. Previously, our results showed that ER stress-mediated apoptosis is important for the regulation of intracellular mycobacteria^11^. A recent report suggests that pathogenic intestinal bacteria enhanced prostate cancer development^29^. Therefore, we suggest that induction of Par-4 protein is a host defense mechanism against mycobacterial infection.

Here, we report that Par-4 production leads to the elimination of mycobacteria via ER stress-mediated apoptosis. Therefore, our results provide insight into new therapeutic strategies for tuberculosis and anti-tumor mechanisms of BCG immunotherapy for prostate cancer.

## Materials and Methods

### Cell culture

Murine macrophage RAW 264.7 cells were grown in Dulbecco’s modified Eagle’s medium (DMEM) supplemented with 10% heat-inactivated fetal bovine serum (FBS), penicillin (100 IU/mL), and streptomycin (100 mg/mL). The cells were cultured in 6-well polypropylene tissue culture plates at 5% CO_2_ and 37 °C for 24 h to ensure cell adherence before infection. Primary BMDMs were obtained from C57BL/6 mice (6–8 weeks old) and cultured in medium containing M-CSF (25 ng/mL; R&D) for 3–5 days to promote differentiation. The human monocyte THP-1 cells and the human prostate cancer PC-3 cells were maintained in RPMI 1640 with 300 mg/L L-glutamine supplemented with 10% FBS, penicillin, and streptomycin. THP-1 cells were incubated with 20 nM phorbol-12-myristate-13-acetate (PMA) to promote differentiation into macrophage-like cells.

### *Mtb* culture and infection

*Mycobacterium tuberculosis* strain H37Ra (ATCC 25177) was cultured in Middlebrook 7H9 liquid medium supplemented with 10% oleic acid, albumin, dextrose, catalase (OADC), and 5% glycerol. Bacteria were resuspended in phosphate-buffered saline (PBS) at a concentration of 1 × 10^8^ CFU/mL. *Mtb* H37Ra was stored at −70 °C until used. The cells were infected with *Mtb* H37Ra at a MOI of 1:1–10:1 for 3 h. To remove non-infected bacteria, cells were washed and cultured with medium containing 5% FBS.

### Chemicals and antibodies

IREstatin (a specific inhibitor of IRE1) was purchased from Axon Medchem (Groningen, the Netherlands). Tunicamycin (TM), salubrinal (eIF2α inhibitor), Z-LEHD-FMK (specific caspase-9 inhibitor), and Z-DEVD-FMK (specific caspase-3 inhibitor) were purchased from Calbiochem (EMD Millipore, Billerica, MA, USA). N-acetyl cysteine (NAC) and cis-Diammineplatinum(II) dichloride (cisplatin) were purchased from Sigma-Aldrich (St. Louis, MO, USA). Z-IETD-FMK (specific caspase-8 inhibitor) was purchased from R&D systems (Minneapolis, MN, USA). LPS was purchased InvivoGen (San Diego, CA, USA). These reagents were diluted in DMEM and cells were pretreated for 30 min–1 h before *Mtb* infection. Dimethylsulfoxide (DMSO) was used as a solvent control. The specific primary antibodies were anti-Par-4 (Cell Signaling Technology, Danvers, MA, USA), anti-GRP78/BiP (Cell Signaling Technology), anti-CHOP (Cell Signaling Technology), anti-phospho-eIF2a (Cell Signaling Technology), anti-caspase-3 (Cell Signaling Technology), anti-β-actin (Santa Cruz Biotechnology, Dallas, TX, USA), anti-GRP78 (Santa Cruz Biotechnology), anti-IRE-1a (Santa Cruz Biotechnology), anti-caspase-9 (Cell Signaling Technology), anti-caspase-8 (Cell Signaling Technology), anti-Lamin B1 (Santa Cruz Biotechnology), anti-beta Tubulin (Abcam), anti-Cytochrome c (BD Pharmingen Inc., San Diego, CA, USA), anti-IκBα (Cell Signaling Technology), anti-phospho-IκBα (Cell Signaling Technology), anti-COX IV (Abcam), anti-Na^+^/K^+^ ATPase (Merck Millipore), and anti-BID (R&D systems). The secondary antibodies were anti-rabbit IgG-HRP (Cell Signaling Technology), anti-mouse-IgG-HRP (Calbiochem), and anti-goat-IgG-HRP (Santa Cruz Biotechnology).

### Reverse transcription-polymerase chain reaction (RT-PCR) analysis

For RT-PCR, total RNA was extracted from RAW 264.7 cells using TRIzol (Invitrogen, Carlsbad, CA, USA). The cDNA was amplified using specific primers for Par-4, GRP78, and β-actin. β-actin was used as control and PCR products were separated on 1.5% agarose gel. The sequences of the primers used for this reaction are as follows:

Par-4-F, 5′-CCAGCGCCAGGAAAGGCAAAG-3′

Par-4-R, 5′-CTACCTTGTCAGCTGCCCAACAAC-3′

GRP78-F, 5′-GTATTGAAACTGTAGGAGGTGTC-3′;

GRP78-R, 5′-TATTACAGCACTAGCAGATCAG-3′;

β-actin-F, 5′-ATCTGGCACCACACCTTCTACAATGAGCTGCG-3′;

β-actin-R, 5′-CGTCATACTCCTGCTTGCTGATCCACATCTGC-3′.

### Western blot analysis

Whole cells were lysed in RIPA buffer (ELPIS biotech, Daejeon, Korea) with a protease inhibitor cocktail. Extracted proteins were resolved by 12% SDS-PAGE and transferred to a polyvinylidene difluoride (PVDF) membrane. Then proteins were blocked with TBS-T containing 5% skim milk (Santa Cruz Biotechnology) at room temperature for 1 h and incubated overnight at 4 °C with the primary antibody (1:1000). The PVDF membrane was washed in TBS-T and incubated for 2 h at room temperature with HRP-conjugated secondary antibody (1:2000). β-actin was used loading control for protein. The bound antibodies were detected using chemiluminescent HRP substrate (ECL, Millipore, Billerica, MA). The blots were exposed to Fuji medical X-Ray film RX-N (Fuji, Japan) or quantified using an Alliance Mini 4M (UVITEC Cambridge, UK).

### Transfection of small interfering RNA

Silencing of Par-4 and GRP78 was carried out by the small interfering RNA technique. The siRNA (200 nM) against mouse Par-4 and negative control siRNAs were purchased from Santa Cruz biotechnology, Inc. (Santa Cruz Biotechnology). siRNA for mouse GRP78 mRNA target sequences was manufactured by Bioneer Corporation (Bioneer Corporation, South Korea). RAW 264.7 cells cultured in 6-well plates were transfected with siPar-4 and siGRP78 using GenMute (SignaGen Laboratories) in accordance with the manufacturer’s recommended protocol. After 5 h of incubation, the cells were cultured with fresh complete medium containing 5% FBS without antibiotics for *Mtb* infection. The harvested cells were used for Western blotting or measuring intracellular survival of *Mtb*.

### Measurement of reactive oxygen species (ROS)

Intracellular superoxide levels were measured using a DCFH-DA assay. RAW 264.7 cells were infected for 0–1 h and then fixed using 4% paraformaldehyde. Next, cells were stained with a 5 μM of Dichlorofluorescin diacetate (DCFH-DA, Molecular Probes, Eugene, OR, USA) for 30 min. Positive cells were identified using a fluorescence microscope Olympus DP70 (400 × magnification).

### Immunofluorescence

RAW 264.7 cells were grown on 18 mm coverslips for 24 h at 37 °C. After *Mtb* infection for 3 h, the cells were fixed using 4% paraformaldehyde, and then washed three times with TBS-T. Next, the cells were blocked with 5% skim milk for 1 h at room temperature. After blocking, cells were cultured with primary antibodies overnight, and then incubated with the appropriate secondary antibody (Alexa Fluor 594 anti-rabbit IgG, Alexa Fluor 594 anti-mouse IgG, Alexa Fluor 488 anti-rabbit IgG, Alexa Fluor 488 anti-mouse IgG, Life technologies) for 2 h at room temperature. Next, the cells were stained with DAPI (0.2 μg/ml) to label DNA. The stained cells were visualized on a fluorescence microscope Olympus DP70 (400 × magnification).

### Cloning

Primers for Par-4 cloning were manufactured by Cosmo Genetech (South Korea). For ligation with the vector, amplified PCR products were incubated for 150 sec at room temperature and 10 min on ice. After ligation, a transformation was performed with *E.coli* Top 10 for 20 min on ice, 90 sec at 42 °C, and 10 min on ice. Then, these products were cultured in LB broth media for 1 h at 37 °C, and centrifuged for 1 min at 13,000 RPM. After removal of the supernatant, suspended cells were incubated for 24 h at 37 °C in LB agar media containing ampicillin (100 μg/mL). The selected colony was cultured in LB broth media containing ampicillin (100 μg/mL) for 24 h at 37 °C. The plasmids were isolated using a plasmid DNA mini-prep kit (ELPIS biotech, Daejeon, Korea) in accordance with the manufacturer’s recommended protocol. Sequence analysis was performed on Cosmo Genetech (South Korea). The RAW 264.7 cells were transfected with the mouse Par-4 expression vector (pcDNA3.1-Par-4) and the empty vector (pcDNA3.1) using Lipofectamine (Invitrogen, Carlsbad, CA), and then these cells were incubated with fresh complete medium containing 5% FBS without antibiotics.

### Statistical analyses

Each experiment was performed at least three times. Statistical significance was analyzed using GraphPad Prism 5 by one-way ANOVA, Friedman’s test, or Wilcoxon signed rank test. Statistical significance was indicated by *p < 0.05, **p < 0.01 and ***p < 0.001.

## Additional Information

**How to cite this article**: Han, J.-Y. *et al.* The Role of Prostate Apoptosis Response-4 (Par-4) in *Mycobacterium tuberculosis* Infected Macrophages. *Sci. Rep.* **6**, 32079; doi: 10.1038/srep32079 (2016).

## Supplementary Material

Supplementary Information
