# Supplementary material for: The Role of Prostate Apoptosis Response-4 (Par-4) in Mycobacterium tuberculosis Infected Macrophages
Source: Sci Rep. 2016 Aug 24;6:32079. doi: 10.1038/srep32079 (PMC4995434; doi:10.1038/srep32079)
Supplement: Supplementary Information [file srep32079-s1.pdf]

The Role of Prostate Apoptosis Response-4 (Par-4) in *Mycobacterium tuberculosis* Infected Macrophages

Supplementary information

Ji-Ye Han, Yun-Ji Lim, Ji-Ae Choi, Jung-hwan Lee, Sung-Hee Jo, Sung-Man Oh and Chang-Hwa Song

Supplementary Figure 1

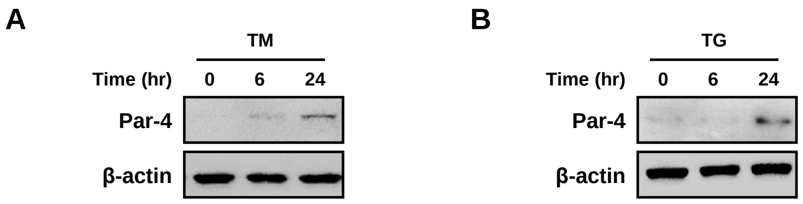

Supplementary figure 1: Par-4 production is associated with ER stress in macrophages. RAW264.7 cells were treated with (a) tunicamycin (2 ug/ml) and (b) thapsigargin (5 uM) for 0-24 h. Western blot analysis was performed using antibodies targeted for Par-4 and β-actin.
